# 2587. Evaluating Seasonality & Demographic Risk Factors of Hospital Encounters in Patients with Community Acquired Pneumonia (CAP)

**DOI:** 10.1093/ofid/ofad500.2202

**Published:** 2023-11-27

**Authors:** Taikchan Lildar, Chineen Shah, Alvi Khan, Nishith Sutaria, Andrew Miele, Aldona Chorzepa, Thomas A Bozzo, Jonathan Robitsek, Javeria Shakil, Kelly L Cervellione

**Affiliations:** Flushing Hospital Medical Center, Flushing, New York; Jamaica Hospital Medical Center, Jamaica, New York; Flushing Hospital Medical Center, Flushing, New York; Jamaica Hospital Medical Center, Jamaica, New York; St Johns University, Queens, New York; St Johns University, Queens, New York; St Johns University, Queens, New York; St Johns University, Queens, New York; Flushing Hospital Medical Center, Flushing, New York; MediSys Health Network, Jamaica, New York

## Abstract

**Background:**

Community Acquired Pneumonia (CAP) is associated with increasing hospitalization rates and is a leading driver of morbidity and mortality. Seasonal differences associated with pathogen incidence in CAP-related hospital admissions have been found (Cilloniz et al., 2017). However, seasonal differences in CAP-related readmissions have not been studied.

**Table 1.**
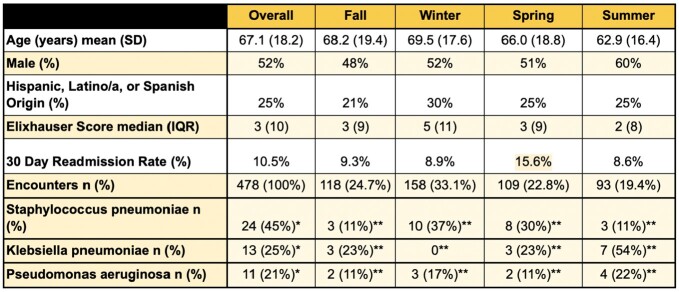
Descriptive characteristics of the sample overall & by season. *percentages calculated as proportion of total pathogens identified, n=53 **calculated as proportion of total for each pathogen

**Figure:**
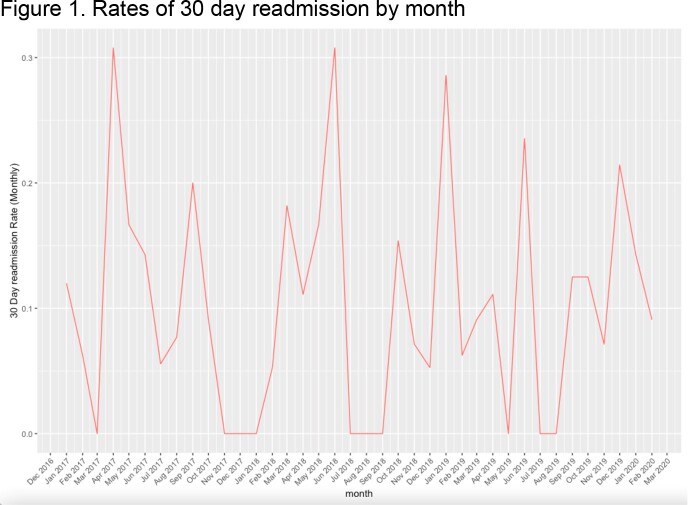

**Methods:**

This retrospective study examined 478 CAP-related admissions from 01/2017-03/2020 within an urban healthcare network in Queens, NY. The primary outcome was rates of readmission at 30 days. Predictors included demographics (e.g., age), clinical factors (e.g., Elixhauser comorbidity score), sputum culture bacteria present, and season of admission (Winter: 21 DEC-20 MAR, Spring: 21 MAR-20 JUN, Summer: 21 JUN-21 SEP, Fall: 22 SEP-20 DEC).

**Results:**

Rates of hospital encounters due to CAP were highest in winter (33%) while rates of 30 day-readmission were highest in the spring (16%). Significant differences in total number of encounters were found by season (58% Winter, 25% Fall, 23% Spring, 19% Summer; p< 0.001) but not readmissions (p=0.29). Sputum culture data was available for 314 patients, with microbial etiology identified in 53 patients. Significant seasonal variations in Klebsiella pneumoniae incidence were found (53% summer, 23% fall & summer, 0% winter, p=0.02). Patients presenting in winter were significantly older than other seasons (avg.=71.5, p=0.01). Across seasons, patients readmitted within 30 days had significantly higher Elixhauser scores (median score=9 vs. median score=3, p=0.003). No significant differences were observed between seasons or readmission in terms of gender, race/ethnicity, smoking history, or Elixhauser score.

**Conclusion:**

Seasonal differences in CAP-related encounters were observed, in line with previous studies. Descriptive differences in rates of readmission suggest that future research about risk factors is needed. This study is limited by its retrospective nature, low sputum culture rates, and unstandardized sputum culture collection methods. Data on viral pneumonia was not collected.

**Disclosures:**

**All Authors**: No reported disclosures

